# Revisiting the Role of Surgery in Non-cirrhotic Portal Hypertension: An Ambispective Observational Study in a Tertiary Care Centre

**DOI:** 10.7759/cureus.99461

**Published:** 2025-12-17

**Authors:** Adarsh Girish, Prabhakaran R, Sugumar Chidambaranathan, Livin Jose J R

**Affiliations:** 1 Surgical Gastroenterology, Madras Medical College, Chennai, IND

**Keywords:** devascularization surgery, non-cirrhotic portal hypertension, proximal splenorenal shunt, rebleeding, variceal rebleeding

## Abstract

Background: Extrahepatic portal vein obstruction (EHPVO) and non-cirrhotic portal fibrosis (NCPF) come under the broad category of non-cirrhotic portal hypertension (NCPH). Current management of this condition primarily focuses on controlling and preventing variceal bleeding through endoscopic and medical therapy, with surgery typically reserved for cases where endotherapy fails. However, the role of surgical interventions, either shunt surgery or splenectomy with esophagogastric devascularization, warrants re-evaluation due to their possible efficacy in controlling variceal bleeding. In addition, surgery addresses other complications associated with NCPH, including symptomatic splenomegaly, hypersplenism, and portal biliopathy.

Methods: In this ambispective single-centre study, patients with NCPH who underwent either proximal splenorenal shunt (PSRS) or splenectomy with esophagogastric devascularization were included. Preoperative data were extracted from electronic medical records, while postoperative follow-up focused on assessing rebleeding episodes, esophagogastroduodenoscopy findings, hematological parameters, and the requirement for additional endoscopic interventions. Optimal outcome was defined as the absence of rebleeding/new-onset bleeding, resolution of varices to grade 0-I, and no postoperative requirement for endoscopic therapy.

Results: Rebleeding or new-onset bleeding occurred postoperatively in only one patient (2.5%) from the entire study population. Overall, optimal outcomes were achieved in 43 patients (74.1%). On subgroup analysis, 21 patients (84%) of the PSRS group and 22 patients (66.6%) who underwent splenectomy with devascularization achieved optimal outcomes. Surgical intervention was associated with significant improvement in hemoglobin, total leukocyte count, and platelet count, along with a marked reduction in variceal grade on follow-up endoscopy.

Conclusion: Surgical management in NCPH is highly effective in preventing rebleeding from esophagogastric varices and yields favorable outcomes in terms of variceal resolution and reduced postoperative need for endoscopic interventions. These findings support the consideration of surgery as a primary management option in selected patients with NCPH and not just in those who have failed medical and endoscopic options.

## Introduction

Non-cirrhotic portal hypertension (NCPH), encompassing extrahepatic portal vein obstruction (EHPVO) and non-cirrhotic portal fibrosis (NCPF), is primarily complicated by upper gastrointestinal bleeding from esophagogastric varices. The Baveno consensus provides widely adopted guidelines for managing these varices, emphasizing non-surgical approaches such as pharmacotherapy with non-selective beta blockers and endotherapy including endoscopic variceal ligation or sclerotherapy. However, data on medical, endoscopic, or surgical management specifically in NCPH remain limited, and treatment is often extrapolated from cirrhotic portal hypertension protocols [1].

NCPH differs from cirrhotic portal hypertension in pathology, hemodynamics, and liver function. NCPF is characterized by phlebosclerosis, periportal and perisinusoidal fibrosis, and the dilation of the main portal vein with thickened walls, often accompanied by thrombosis in medium and small portal vein branches [2-4]. EHPVO represents the obstruction of the extrahepatic portal vein, with or without the involvement of intrahepatic radicles. Both conditions preserve lobular liver architecture and normal liver function, unlike cirrhosis. Hemodynamically, vascular resistance occurs at the prehepatic level in EHPVO and at pre- and perisinusoidal levels in NCPF, while cirrhosis involves sinusoidal resistance, resulting in elevated hepatic venous pressure gradient (HVPG); in contrast, HVPG remains normal in EHPVO and near-normal in NCPF [4,5].

Medical and endoscopic therapies, although effective for variceal bleeding, do not address other complications of NCPH, including symptomatic splenomegaly, hypersplenism, growth retardation, or portal biliopathy, which may coexist with varices. Given the lack of high-quality studies for NCPH-specific guidelines and the limitations of non-surgical management, the role of surgical intervention warrants further investigation. Research is needed to determine whether surgical management should be considered a primary therapeutic option rather than a last resort.

Surgical strategies include shunt surgery and esophagogastric devascularization. Shunt surgery is preferred when anatomy permits, while devascularization is reserved for cases with unfavorable anatomy, such as extensive mesenteric venous thrombosis or inadequate vein size. Shunt surgery diverts portal blood flow to reduce variceal rebleeding, reverse hypersplenism, decompress peri-choledochal collateral vessels to relieve portal biliopathy, promote growth in pediatric patients, and prevent bleeding from ectopic varices. Shunts may be non-selective, partially selective, or selective, with the proximal splenorenal shunt (PSRS) being a common non-selective option [6,7].

Esophagogastric devascularization procedures include several techniques, with the Sugiura-Futagawa and Hassab procedures being the most recognized. Hassab's procedure involves gastroesophageal devascularization and splenectomy, including peri-hiatal devascularization of the lower esophagus, separation of the stomach from its bed, ligation of the left gastric artery at the lesser curvature, devascularization of the greater curvature, and suction drainage of the splenic bed [8]. The modified Hassab procedure limits devascularization to the proximal half of the stomach and distal three to four inches of the esophagus, ligates only the ascending branch of the left gastric artery, and avoids opening the esophagus and stomach [9].

This study aimed to measure the postoperative rebleeding rates, esophageal varices resolution/downgrading, and improvement in hemodynamic parameters (hemoglobin, total leukocyte count, and platelet count) in NCPH patients following PSRS surgery or splenectomy with esophagogastric devascularization using the modified Hassab technique.

## Materials and methods

Study design

This was a single-centre (Institute of Surgical Gastroenterology, Madras Medical College, Chennai, India) observational study conducted ambispectively during a study period of nine years and nine months (from January 2016 to September 2025).

In this ambispective study, the data of 49 patients (84%) was collected retrospectively, while that of nine patients (16%) was collected prospectively (starting from February 2025).

Participants and subjects

Study participants included all EHPVO and NCPF patients who underwent either PSRS surgery or splenectomy with esophagogastric devascularization by the modified Hassab procedure during the stipulated period. Patients with cirrhosis and those who were lost to follow-up (with a minimum of three months) after surgery were excluded from the study.

Data collection

Data on preoperative variables were collected from the institute's electronic databases, including operating theatre logs, intraoperative notes, discharge summaries, inpatient case sheets, and investigation charts, which contained all relevant preoperative blood tests and imaging reports. When additional information was required, patients were contacted over the telephone or reviewed in an outpatient setting to obtain a detailed history.

For patients operated on after the start of the study, data were collected prospectively through direct history taking, as well as from inpatient case sheets and investigation charts. The preoperative variables recorded included age at the time of surgery, sex, indication for surgery, number of prior bleeding episodes, preoperative esophagogastroduodenoscopy (EGD) findings, number of preoperative endoscopic interventions (including endoscopic variceal ligation or sclerotherapy), and preoperative complete blood counts (hemoglobin, total leukocyte count, and platelet count).

The primary indication for surgery was bleeding refractory to endoscopic therapy, whereas secondary indications included symptomatic splenomegaly, symptomatic hypersplenism, or portal biliopathy. Preoperative EGD was performed for all patients during the same hospital admission prior to surgery. EGD findings were categorized according to the grade of the largest varices and the presence of isolated gastric varices, using the Westaby classification [10]. Preoperative complete blood counts included in the study were those performed during hospital admission prior to preoperative optimization or blood transfusion for surgery.

Postoperatively, patients were followed on an outpatient basis through history taking, repeat blood investigations (including complete blood counts and liver function tests), and EGD performed at least three months after surgery. Postoperative variables included the number of postoperative rebleeding episodes, variceal grade based on follow-up EGD, postoperative complete blood counts (hemoglobin, total leukocyte count, and platelet count), the number of postoperative endoscopic interventions required for persistent or recurrent varices, and the modified Hepatocyte Activity Index (HAI) in cases where intraoperative liver biopsy was obtained.

Statistical analysis

Data entry and preliminary data cleaning were performed using Microsoft Excel (Microsoft Corp., Redmond, WA, USA), and statistical analysis was done using IBM SPSS Statistics for Windows, V. 16.0 (SPSS Inc., Chicago, IL, USA). Continuous variables were expressed as mean±standard deviation (SD) and categorical variables as frequencies and percentages. Associations between groups were made using the chi-squared test for categorical variables. Pre- and post-comparisons were made using the Wilcoxon signed-rank test for continuous variables. A p-value of <0.05 was considered statistically significant.

Approval

The study was approved by the Institutional Ethics Committee of Madras Medical College (approval number: IEC-MMC/Approval/10022025). 

## Results

In this study, we assessed the outcomes of NCPH patients who underwent either PSRS surgery or splenectomy with esophagogastric devascularization.

A total of 58 (100%) NCPH patients were included in this study; 26 patients (44.8%) had NCPF, while 32 patients (55.2%) were diagnosed as EHPVO (Table 1).

**Table 1:** Baseline parameters of the study patients (n=58; 100%) NCPF: non-cirrhotic portal fibrosis; EHPVO: extrahepatic portal vein obstruction

Parameter	Value
Median age (years)	27
Males	24 (41.3%)
Females	34 (58.6%)
NCPF patients	26 (44.8%)
EHPVO patients	32 (55.2%)
Bleeders	40 (69%)
Non-bleeders	18 (31%)

Out of these, 40 patients (69%) were bleeders with at least one episode of upper gastrointestinal bleeding from the varices. All bleeders had been on a combination of pharmacotherapy and endotherapy (endoscopic variceal ligation or endoscopic sclerotherapy) with an average of two preoperative endoscopic variceal ligation or endoscopic sclerotherapy sessions in the bleeders. The remaining 18 patients (31%) were non-bleeders.

Preoperatively, EGD was done and the esophagogastric varices were graded as per the Westaby classification and patients with isolated gastric varices were separately noted (Table 2 and Table 3).

**Table 2:** Preoperative EGD findings of bleeders (n=40; 100%) EGD: esophagogastroduodenoscopy; EVL: endoscopic variceal ligation; IGV: isolated gastric varices

Grade of varices prior to surgery	Number of patients
No varices	4 (10%)
Grade I	5 (12.5%)
Grade II	10 (25%)
Grade III	18 (45%)
IGV	3 (7.5%)

**Table 3:** Preoperative EGD findings of non-bleeders (n=18; 100%) EGD: esophagogastroduodenoscopy; EVL: endoscopic variceal ligation; IGV: isolated gastric varices

Grade of varices prior to surgery	Number of patients
No varices	2 (11.1%)
Grade I	5 (27.7%)
Grade II	4 (22.2%)
Grade III	6 (33.3%)
IGV	1 (5.5%)

The primary indications for surgery were as follows: 34 patients (58.6%) had prior failed endotherapy, 16 patients (27.5%) had symptomatic splenomegaly, four patients (6.8%) had symptomatic hypersplenism, and another four patients (6.8%) had symptomatic portal biliopathy (Table 4).

**Table 4:** Primary indications for surgery (n=58; 100%)

Indication	Number of patients
Failed endotherapy	34 (58.6%)
Symptomatic splenomegaly	16 (27.5%)
Symptomatic hypersplenism	4 (6.8%)
Symptomatic portal biliopathy	4 (6.8 %)

In this cohort, 25 patients (43.1%) underwent PSRS surgery, while 33 patients (56.8%) underwent splenectomy with esophagogastric devascularization using the modified Hassab procedure. Splenectomy with devascularization was primarily performed in patients whose intraoperative anatomy precluded PSRS, including those with small splenic vein size or extensive thrombosis of the portal or splenic veins (Table 5).

**Table 5:** Procedure (n=58; 100%) PSRS: proximal splenorenal shunt

Procedure	Number of patients
PSRS	25 (43.1%)
Splenectomy with esophagogastric devascularization	33 (56.8%)

The median follow-up period post-surgery in this study was 36 months, ranging from five months to 113 months.

Among the bleeders, postoperative rebleeding occurred in only one patient (2.5%) over a median follow-up period of 36 months, while none of the non-bleeders developed new-onset variceal bleeding during follow-up. 

When outcomes were stratified by surgical procedure, rebleeding/new-onset bleeding was observed only in a single patient (3.03%) in the splenectomy with devascularization group, whereas no patients in the PSRS group experienced rebleeding. This difference was not statistically significant (p=1.0), indicating that both surgical approaches are comparably effective in preventing postoperative variceal bleeding (Table 6).

**Table 6:** Comparing the rebleeding rates of devascularization vs PSRS PSRS: proximal splenorenal shunt

	Rebleeding +	Rebleeding -	Total	Chi-squared value	P-value by Fisher's exact test
Splenectomy devascularization	1 (3.03%)	32 (96.9%)	33 (100%)	0.77	1.0
PSRS	0 (0%)	25 (100%)	25 (100%)		
Total	1 (1.7%)	57 ( 98.2%)	58 (100%)	-	-

Postoperatively, EGD was repeated in all patients, and varices were graded as per the Westaby classification. Overall, only six patients (10.3%) required repeat endoscopic variceal ligation: five (8%) for large varices detected on follow-up EGD and one (1.7%) due to rebleeding (Table 7 and Table 8).

**Table 7:** Postoperative EGD findings in patients undergoing PSRS (n=25) EGD: esophagogastroduodenoscopy; EVL: endoscopic variceal ligation; IGV: isolated gastric varices

Postoperative EGD (≥3 months post-surgery)	Number of patients
No varices	14 (56%)
Grade I	7 (28%)
Grade II	3 (12%)
Grade III	1 (4%)
IGV	0 (0%)
Postoperative EVL after PSRS	1 (4%)

**Table 8:** Postoperative EGD findings in patients undergoing splenectomy with esophagogastric devascularization (n=33) EGD: esophagogastroduodenoscopy; EVL: endoscopic variceal ligation; IGV: isolated gastric varices

Postoperative EGD (≥3 months post-surgery)	Number of patients
No varices	10 (30.3%)
Grade I	12 (36.3%)
Grade II	5 (15.1%)
Grade III	6 (18.1%)
IGV	0 (0%)
Portal hypertensive gastropathy	5 (15.1%)
Postoperative EVL after splenectomy/devascularization	5 (15.1%)

Preoperative evaluation revealed that 38 patients (65.5%) had grade II or III esophageal varices, whereas postoperative assessment demonstrated a substantial reduction, with only 15 patients (25.8%) exhibiting grade II/III varices (p<0.0001) (Table 4). Additionally, isolated gastric varices were present in four patients (6.8%) prior to surgery; however, no patients demonstrated isolated gastric varices postoperatively (p=0.12) (Table 9 and Table 10).

**Table 9:** Comparing the total percentage of patients with preoperative varices of grades II and III vs the total percentage of patients with postoperative varices of grades II and III

	Grades II+III	Total	Chi-squared value	P-value by the chi-squared test	OR (95% CI)
Preoperative	38 (65.5%)	58 (100%)	18.38	<0.0001	0.18 (0.08-0.41)
Postoperative	15 (25.8%)	58 (100%)			

**Table 10:** Comparing the percentage of isolated gastric varices preoperative vs postoperative IGV: isolated gastric varices

	IGV +	IGV -	Total	Chi-squared value	P-value by Fisher's exact test
Preoperative	4 (6.8%)	54 (93.1%)	58 (100%)	4.14	0.12
Postoperative	0 (0%)	58 (100%)	58 (100%)		

To specifically evaluate the effect of surgery on variceal management, postoperative outcomes were categorized as either optimal or suboptimal. This classification was based on three parameters: the occurrence of postoperative rebleeding or new-onset bleeding, postoperative endoscopic findings, and the need for any endoscopic intervention after surgery. An optimal outcome was defined as the absence of rebleeding or new-onset bleeding, postoperative varices of less than grade II on endoscopy, and no requirement for endoscopic intervention. Any outcome that did not meet these criteria was considered suboptimal.

In this cohort, 43 patients (74.1%) achieved an optimal outcome, whereas 15 patients (25.8%) experienced a suboptimal outcome. Subgroup analysis revealed that 21 patients (84%) in the PSRS group achieved optimal outcomes, compared to 22 patients (66.6%) in the splenectomy with esophagogastric devascularization (p=0.135), suggesting a trend toward better variceal control with PSRS (Table 11).

**Table 11:** Outcomes PSRS: proximal splenorenal shunt

	Optimal outcome	Suboptimal outcome	Total	Chi-squared value	P-value by the chi-squared test
Splenectomy devascularization	22 (66.6%)	11 (33.3%)	33 (100%)	2.229	0.135
PSRS	21 (84%)	4 (16%)	25 (100%)		
Total	43 (74.1%)	15 (25.8%)	58 (100%)		

Postoperatively, there was a significant improvement in hematological parameters (Table 12).

**Table 12:** Comparing hematological parameters preoperative vs postoperative

		Mean	Median	Std. deviation	Minimum	Maximum	P-value by the Wilcoxon signed-rank test
Hemoglobin (g/dL)	Preoperative	9.5	9.5	1.6	6.7	13.6	<0.0001
	Postoperative	11.4	11.2	1.3	6.4	14.0	
Total leukocyte count (/mm³)	Preoperative	3751.7	2800	2170.9	1200	10200	<0.0001
	Postoperative	7303.1	7400	2153.3	4300	13000	
Platelet count (/mm³)	Preoperative	69913.8	61000	42853.2	17000	260000	<0.0001
	Postoperative	400000	340000	170000	190000	1100000	

The median hemoglobin level increased from a preoperative value of 9.5 g/dL to 11.2 g/dL (p<0.0001). Similarly, the median total leukocyte count rose from 2800/mm³ preoperatively to 7400/mm³ postoperatively (p<0.0001). The median platelet count showed a marked increase from 61000/mm³ before surgery to 340000/mm³ after surgery (p<0.0001).

Of the four patients who had preoperative symptomatic portal biliopathy as an indication for surgery, all four of them had preoperative endoscopic retrograde cholangiopancreatography (ERCP) stenting done, and postoperatively, bilirubin levels normalized after stent removal in three patients (75%), while one (25%) patient who had developed extrahepatic biliary stricture had to undergo Roux-en-Y hepaticojejunostomy eight months later. 

For patients with preoperative images reporting altered echoes of the liver or increased stiffness in FibroScan, an intraoperative wedge biopsy of the liver was taken. The biopsy was reported using modified HAI. Modified HAI grading has a score ranging from 0 to 18 depending upon periportal or peri-septal interface hepatitis, confluent necrosis, focal lytic necrosis/apoptosis, and portal inflammation. Among the 30 patients for whom intraoperative liver biopsy was taken, 77.2% of the patients with modified HAI less than or equal to 2/18 had an optimal outcome compared to only 25% in patients with modified HAI greater than 2/18 (p=0.0207) (Table 13).

**Table 13:** Modified HAI and correlation with surgical outcomes HAI: Hepatocyte Activity Index

	Optimal outcome	Suboptimal outcome	Total	Chi-squared value	P-value by Fisher's exact test
HAI ≤2/18	17 (77.2%)	5 (22.7%)	22 (100%)	6.903	0.0207
HAI >2/18	2 (25%)	6 (75%)	8 (100%)		

## Discussion

NCPH is classified as a vascular liver disorder and differs from cirrhotic portal hypertension in terms of etiopathogenesis, hemodynamics, and liver function. Hence, extrapolating cirrhotic portal hypertension management guidelines to NCPH patients may result in suboptimal outcomes [11]. At present, most consensus guidelines reserve surgery for NCPH patients who fail endotherapy. This study focused on the outcomes of surgical management to reassess its role in these patients.

In our study, the rebleeding rate following surgical management was 1.7% (0% in the PSRS group and 3.3% in the devascularization group), and none of the non-bleeders experienced new-onset bleeding during follow-up. These findings align with a recent prospective study from a tertiary care centre in Eastern India, which reported similarly low rebleeding rates among NCPH patients undergoing shunt or devascularization procedures [12]. Another study in schistosomiasis-related NCPH patients undergoing devascularization alone showed a higher rebleeding rate of 14.6% [13].

Although endotherapy is commonly used for variceal management, high-level evidence supporting its efficacy in NCPH patients is limited. Some retrospective studies reported that endoscopic management required multiple sessions per week, with up to five sessions needed in certain cases [14,15]. Recurrence after variceal eradication occurred in 40% of patients [16], and rebleeding rates ranged from 9.2% to 11.9% [15,17]. Therefore, variceal eradication alone may be insufficient and requires frequent endoscopic follow-up, which can be challenging, particularly for patients from lower socioeconomic backgrounds [1,18].

Another drawback of endotherapy is the potential development of isolated gastric varices and portal hypertensive gastropathy after esophageal variceal eradication [19]. In contrast, no such complications were observed following surgical management in our study. Moreover, there was a marked reduction in higher-grade (II/III) varices postoperatively compared to preoperative findings, consistent with results reported by Gopal et al. [20].

Surgical management also effectively addressed other NCPH-related symptoms. Patients operated for symptomatic splenomegaly, hypersplenism, or portal biliopathy experienced resolution of symptoms. Correction of hypersplenism was reflected in improved hematological parameters, with increases in median white blood cell (WBC) count, platelet count, and hemoglobin, aligning with findings from Saluja et al. [21].

Among patients who underwent intraoperative liver biopsy, those with a modified HAI >2/18 had comparatively suboptimal outcomes, suggesting that the modified HAI may have a prognostic role in assessing surgical outcomes.

There are a few limitations to this study. Being retrospective, surgeries were performed by multiple surgeons, potentially resulting in variations in the extent of devascularization, which could have influenced outcomes. Similarly, although varices were graded using the Westaby classification, subjective variation in endoscopic assessment may have occurred. Postoperative Doppler ultrasound was not routinely performed in PSRS patients, so shunt patency and its impact on recurrent varices and rebleeding could not be assessed [22].

Despite these limitations, the results suggest that surgery is a safe and effective alternative for secondary prophylaxis of variceal bleeding and may even be considered as a first-line treatment option in NCPH patients.

## Conclusions

Our study demonstrates that surgical management, through either PSRS surgery or esophagogastric devascularization with splenectomy, is highly effective in controlling variceal bleeding in patients with NCPH. In addition to preventing rebleeding, these surgical approaches comprehensively address other complications of NCPH, including symptomatic splenomegaly, hypersplenism, and the less common presentations such as portal biliopathy. Compared to current standard practices involving medical therapy or endoscopic interventions, surgery may provide a more definitive solution tailored to the unique pathophysiology of NCPH. Nonetheless, further high-quality studies are warranted to refine management strategies and optimize outcomes for this patient population.
